# C1QBP suppresses cell adhesion and metastasis of renal carcinoma cells

**DOI:** 10.1038/s41598-017-01084-w

**Published:** 2017-04-20

**Authors:** Yong Wang, Donghe Fu, Jing Su, Yajing Chen, Can Qi, Yin Sun, Yuanjie Niu, Ning Zhang, Dan Yue

**Affiliations:** 1 Department of Urology, Tianjin Medical University Second Hospital, Tianjin Institute of Urology, Tianjin Medical University, Tianjin, 300211 China; 2 School of Laboratory Medicine, Tianjin Medical University, Tianjin, 300203 China; 3 Department of Urology, Children’s Hospital of Hebei Province, Shijiazhuang, 050031 China; 4 Department of Radiation Oncology, University of Rochester Medical Center, Rochester, NY 14642 USA; 5 Tianjin Medical University Cancer Institute and Hospital, National Clinical Research Center for Cancer, Key Laboratory of Cancer Prevention and Therapy, Research Center of Basic Medical Sciences, Tianjin, 300070 China

## Abstract

Complement component 1q subcomponent binding protein (C1QBP) is a ubiquitously expressed cellular protein and can be upregulated or activated in a variety of malignant tumors, including those from thyroid, colon and breast, but its role remains unclear in renal cell carcinoma (RCC). In this study, C1QBP knockdown in RCC cell influenced expression of multiple genes associated with cell adhesion, among which L1 cell adhesion molecule (L1CAM) was significantly higher upon a reduction of C1QBP. In turn, cell adhesion and invasion abilities were significantly increased with increased metastasis to lung and liver *in vivo*. C1QBP may regulate RCC cell adhesion and invasion through influencing the p-GSK3/β-Catenin/L1CAM expression. Over all, our study demonstrated that C1QBP could regulate RCC metastasis by regulating the GSK3/β-Catenin/L1CAM signaling pathway.

## Introduction

Complement component 1q subcomponent binding protein (C1QBP) also known as HABP1, p32 and gC1qR, is a ubiquitously expressed, multi-ligand-binding, multicompartmental cellular protein involved in various ligand-mediated cellular responses^1^. It is widely present on mitochondria, nucleus, cytoplasm, Golgi apparatus and cell membranes, and can be secreted into extracellular matrix. It may be involved in intracellular molecular transport, connecting different organelles and the signal transduction between organelles and the cell membrane. Sequence analysis of this gene has further revealed that C1QBP is synthesized as a precursor protein of 282 amino acids which undergoes a post-translational modification to give rise to the mature form of 209 amino acids by proteolytic cleavage of 73 amino acids at the N-terminus^2^. The role of C1QBP in cell-adhesion is well established and in combination with HA, it might facilitate the process of adhesion and de-adhesion during mitotic stages^3^. Interaction of C1q with endothelial cells and platelets, for example, leads to cellular activation followed by release of biological mediators and/or expression of adhesion molecules^4^. C1QBP can also function as an ASF/SF2 inhibitory factor, regulating ASF/SF2 RNA binding and phosphorylation^5^.

Enhanced C1QBP expression has been reported in adenocarcinomas arising in a variety of organs^6^, for example in the thyroid, colon, pancreas, stomach, esophagus, and lung^7^. C1QBP is found to be essential for cancer cell chemotaxis and metastasis, its expression level is closely linked with distant metastasis and TNM stages in breast cancer^8^, and there is a positive correlation between the expression of C1QBP and Gleason score, pathologic stage, tumor recurrence in prostate cancer patients. But it has also been documented that C1QBP is significantly decreased in human cervical squamous cell carcinoma tissues relative to normal cervix tissues and inhibits viability, migration and proliferation of cervical squamous cells carcinoma via the p38 MAPK signaling pathway^9^. Consistent with the finding in cervical squamous cell carcinoma, our study^10^ shows that the level of C1QBP in the renal carcinoma tissues is significantly lower than that in the adjacent normal tissues, and the expression of C1QBP could be used as an independent prognostic maker for cancer progression in the renal cell carcinoma (RCC) patients. In a word, it is tissue specificity of C1QBP expression and C1QBP appears to be tumor suppresser in RCC. To examine the detailed mechanism of the C1QBP function in RCC, we carried out *in vitro* and *in vivo* studies investigating the role of C1QBP in spontaneous metastasis of RCC cells with a focus on RCC adhesion and metastasis.

## Materials and Methods

### Cell culture

Renal cell carcinoma 786-0, ACHN and HEK-293T cell lines were obtained from American Type Culture Collection. Cells were cultured in DMEM (Hyclone) supplemented with 10% fetal bovine serum (Hyclone) and 1% glutamine Pen-Strep solution (BI) at 37 °C in an incubator with 5% CO_2_.

### Plasmid construction and cell transfection

According to *C1QBP* mRNA in GeneBank, a human *C1QBP* shRNA interference sequence was synthesized, and a scramble sequence has same GC content as the target site was designed. Both of them were linked to lentiviral vector pLKO.1-TRC (Addgene, USA) and generated recombinant plasmids pLKO.1-shC1QBP and negative control pLKO.1-scr. After gradient annealing of oligo, pLKO.1-TRC was linearized by *Age I* (TaKaRa) and *EcoR I* (TaKaRa), the target vector segment was obtained by DNA agarose (BIOWEST) gel electrophoresis. Annealing product shDNA and scrDNA were linked with pLKO.1 by T4 DNA ligase (TaKaRa) at 16 °C overnight, and then transformed into *DH5a* (Beijing Tian Gen biological co., LTD). After plasmids were extracted, they were identified by *EcoRI*, *NcoI* (TaKaRa) digested and PCR, finally the sequencing identification were done by company (TaKaRa) to make sure lentiviral vector pLKO.1-shC1QBP and pLKO.1-Scr were successfully constructed. HEK-293T cell were transfected using Lipofectamine 2000 (Invitrogen) to obtain lentivirus particles, 786-0 and ACHN were transfected by lentivirus, stable cell lines were selected by using puromycin (2 μg/ml, Beijing, China biological technology liability co., LTD) and further expanded in the presence of puromycin (0.5 μg/ml) containing medium. The expression of C1QBP was confirmed by Western blotting, and membranes were visualized with GBOXI Chemi XT Imaging System (SYNGENE), and real-time PCR with 7500 Fast (ABI).

### Total RNA extraction and microarray assay

Total mRNA was extracted from the cells using Trizol Reagent (Invitrogen). Samples were sent to Jingtai Bio-tech company (Shanghai, China) for miRNA isolation, quality control, chip hybridization, and microarray data analysis, the samples were purified according to the manufacturer’s instructions (QIAGEN, Valencia, CA), cDNA was synthesized with SuperScript II (Invitrogen), and then purified with RNeasy Mini Kit (QIAGEN). Labeled with biotin and hybridized at 45 °C for 16 h to Affymetrix GeneChip Human Gene 1.0 ST arrays (Affymetrix). For each sample, three biological replicates were performed. All arrays were washed and scanned using a GeneChip Scanner 3000 (Affymetrix) at correct pixel value (3 um) and wavelength (570 nm), and data were collected and analysised. Genes expressed differentially with at least 2-fold change with *P* < 0.05 in either direction were considered as up or down regulated.

### Quantitative real-time polymerase chain reaction (Real-time PCR)

Total RNA was extracted from cells using the Trizol reagent (Invitrogen, Carlsbad, CA, USA). Then 1 μg total mRNA of each sample was used for reverse transcription with Omniscript RT kit (Qiagen, Hilden, Germany). Real-time PCR was performed with a Roche FastStart Universal SYBR Green Master kit (Roche, Switzerland), according to the manufacturer’s instructions. The primer sequences were shew in Table 1. Relative expression was normalized and the data was analyzed by the 2^−ΔΔCt^ method.

**Table 1:** Primer sequences.

Gene		Primer sequences
C1QBP	Former primer	AGTGCGGAAAGTTGCCGGGGA
	Reverse primer	GAGCTCCACCAGCTCATCTGC
CLDN4	Former primer	AGCTCTGTGGCCTCAGGACTCT
	Reverse primer	CAGTGATGAATAGCTCTTCTTAAATTACAA
MPZL3	Former primer	GAGCAGGTCTGGCTATAAGAAGTC
	Reverse primer	GAATGGGATGTTTCCTGTCTTTAG
Rnd-3	Former primer	AATAGAGTTGAGCCTGTGGG
	Reverse primer	CTAATGTACTAACATCTGTCCGC
Cadherin-11	Former primer	ACCCTCACCATCAAAGTCTG
	Reverse primer	TCAGGGTCACAAACAATACT
CTGF	Former primer	CAGAACCACCACCCTGCCG
	Reverse primer	CGTACATCTTCCTGTAGTACA
L1CAM	Former primer	ACGAGGGATGGTGTCCACTTCAAA
	Reverse primer	TTATTGCTGGCAAAGCAGCGGTAG
CCDC80	Former primer	CCTGGGCAGCGAGAAGAAGAAAG
	Reverse primer	CCGGGATGGAGGGTAAAGATT
hFat	Former primer	TATCACAAAACGCCCTTGCT
	Reverse primer	TGGATTGTCATTGATATCCTG
IL-8	Former primer	ATGACTTCCAAGCTGGCCGTGGCT
	Reverse primer	TCTCAGCCCTCTTCAAAAACTTCTC
Del1	Former primer	AAGTGAAAGGCACCAATG
	Reverse primer	CTCAGAACAACCCGACAG
GAPDH	Former primer	TGCACCACCAACTGCTTAGC
	Reverse primer	GGCATGGACTGTGGTCATGAG

### Cell adhesion Assay

Fibronectin (10 μg/ml, Sigma) was used to coat the cover glass in 35 mm culture dish, ultraviolet irradiate overnight, washed 3 times with sterile purified water. Cells were resuspended in serum-free medium (2.7 × 10^5^/ml) and then plated in 35 mm dishes containing coated cover glass. After 5 min, 15 min and 30 min of incubation, cells were washed gently twice with cold PBS and then fixed with 4%PFA (Solarbio). Cells attached to the coverslips were stained with crystal violet and counted under a microscope (Olympus, Japan) with five random fields.

### Cell migration assay

Transwell with 8.0 μm pore polycarbonate membrane insert and 1 × 10^5^ pores per cm^2^ (Corning, Inc., Corning, NY) was used. RCC cells (1 × 10^4^ cells/well) were harvested and seeded with serum-free DMEM medium into the upper chamber, and the lower chamber contained DMEM medium with 10% FBS. After 6 h incubation at 37 °C, the invaded cells were fixed with paraformaldehyde and stained with crystal violet. Cell numbers were counted in five randomly chosen microscopic fields.

### Cell invasion assay

The invasion capabilities of RCC cells were determined by the transwell assays. The upper transwell chambers containing 8 μm-pore-size polycarbonate membrane filters (Corning Inc., Corning, NY, USA) were pre-coated with diluted growth factor-reduced matrigel (BD, Inc) (1:5 serum free RPMI, 50 µg/chamber) and put into the incubator for 5 h. RCC cells were then harvested and seeded with serum-free DMEM media into the upper chamber at 1 × 10^5^ cells/well, and the bottom chambers contained DMEM with 10% FBS, and then transwells were incubated for 48 h at 37 °C. Following incubation, the invaded cells attached to the lower surface of the membrane were fixed using 4% paraformaldehyde and stained with 1% crystal violet. Cell numbers were counted in 6 randomly chosen microscopic fields (100×) per membrane.

### 3D invasion assay

The 3D invasion assay was modified from the previous report^11^. Adherent cells should be cultured to no greater than 80% confluence. Collagen mix (collagen 500 ul; 10X PBS 63 ul; 0.1 M NaOH 63 ul; 0.1 M HCl 12.5 ul) and Matrigel (10 mg/ml) were mixed (ratio = 1:1) and added in 6-well plates. Harvest and count cells, 1 × 10^4^ of cells in 200 μl medium containing 1% Matrigel was plated into the collagen/Matrigel coated 6-well plates. The medium was replenished every 3 d. After 10 d incubation, cells with/without protrusions were captured in 6 different random fields.

### Western blotting

Cells were lysed in cold lysis buffer, total protein was extracted, and concentration was measured by BCA (Thermo) method. Protein (40 μg) was separated by SDS-PAGE, then transfer to a PVDF membrane (Millipore). The membrane was blocked with skim milk (5%, BD) or BSA (5%, Solarbio) in TBS containing Tween20 (0.1%) for 1 h, and incubation with primary antibodies β-Catenin (Epitomics, #1247-1), p-GSK-3β (Cell Signaling, #5558), GSK-3β (Cell Signaling, #9315), C1QBP (Santa Cruz, sc-48795), L1CAM (Abcam, ab123990) and β-actin (Santa Cruz, sc-47778) at 4 °C overnight. Finally, the membrane was washed and incubated with secondary antibodies anti-mouse or anti-goat IgG (Santa Cruz) conjugated with horse radish peroxidase for 1 h, and developed by ECL Western Blotting Detection Reagents (Millipore).

### *In vivo* metastasis studies

Twelve mice (6–8 weeks old nude mice) were divided into 2 groups (n = 6, Male: Female = 1:1). We prepared xenografts by an incision in the back of mice and exposure of the left kidney. The mice were injected with 1 × 10^6^ cells (mixture with Matrigel, 1:1) into the sub-renal capsule and incision closed. Cells were also transduced with Luciferase so that tumor in the mice could be measured using a Fluorescent Imager (IVIS Spectrum, Caliper Life Sciences, Hopkinton, MA) at 8 weeks after injection. After the last monitoring with the Imager, mice were sacrificed and primary tumors and metastasis sites were further examined by IHC staining. Procedures involving animals and their care were conducted in conformity with NIH guidelines (NIH Pub. No. 85-23, revised 1996) and was approved by Animal Care and Use Committee of the Tianjin Medical University.

### Immunohistocytochemistry

Paraffin-processed sections with 5 µm thickness were mounted on polylysine-coated glass slides. Slides were dewaxed in 100% xylene and rehydrated by incubation in decreasing concentrations of alcohol, and incubated in 3% H_2_O_2_ to eliminate endogenous biotin. Sections blocked with horse serum were incubated with C1QBP antibody (Santa Cruz, CA) overnight at 4 °C. After being washed with PBS, the immunoreactions were performed using the Max Vision HRP-Polymer anti-Rabbit IHC Kit (Miaxim.bio, China). Sections were developed by peroxidase substrate DAB Detection Kit (Miaxim.bio, China) and were counterstained by hemaoxylin. PBS was used in place of the primary antibodies in the negative controls.

### Statistical analysis

Results were expressed as mean ± standard deviation (SD). Comparison was performed using ANOVA and t-test by SPSS 17.0 software. Value of *P* ≤ 0.05 was considered significant.

## Results

### Gene microarray analysis for RCC cells with C1QBP knockdown

**Figure 1:**
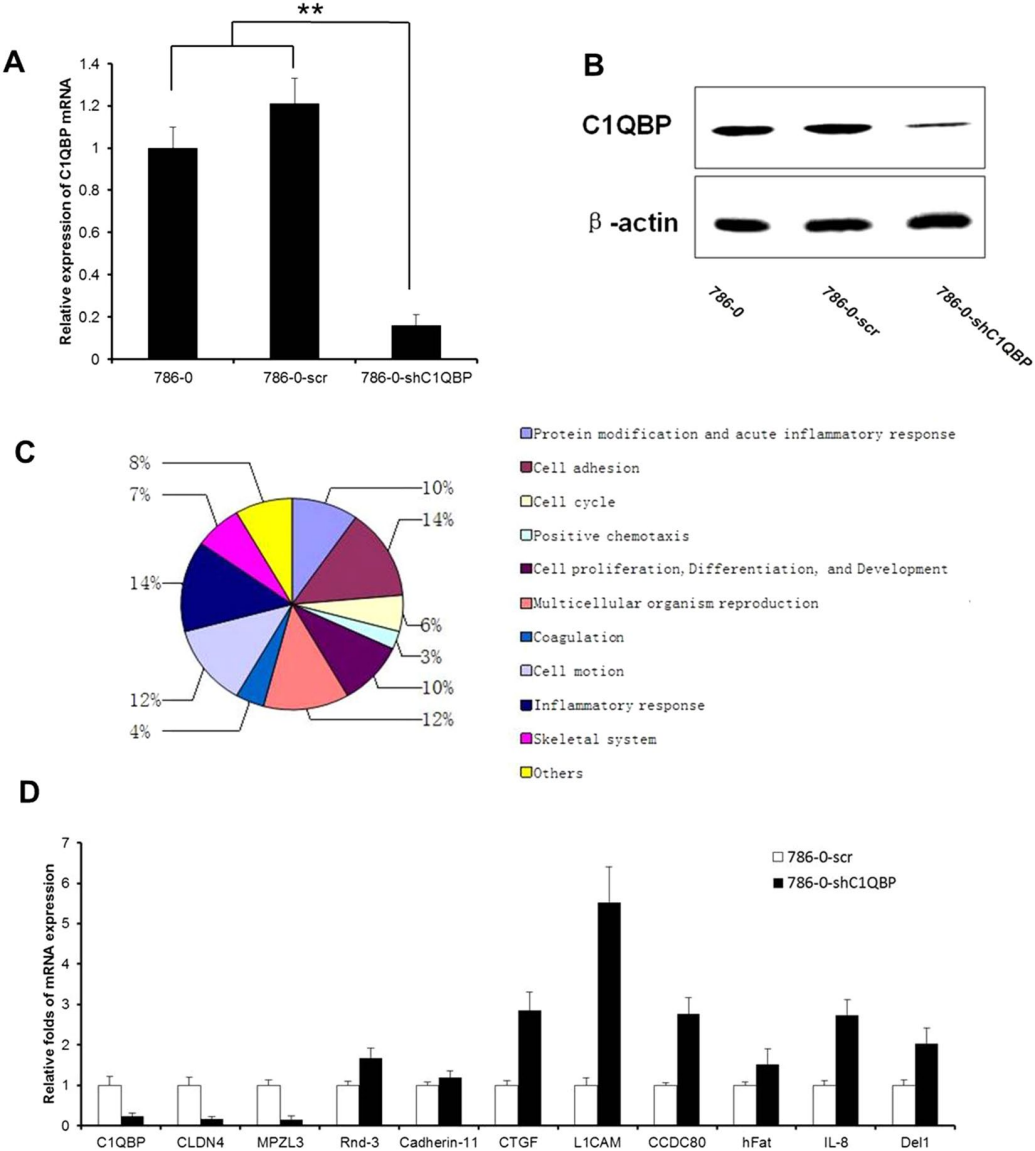
Screening and validation of differentially expressed genes after C1QBP knockdown. (**A**) Total RNA was isolated from 786-0, 786-0-scr and 786-0-shC1QBP, real-time PCR using specific primers for the indicated transcripts revealed the expression of C1QBP after lentivirus transfection. (**B**) C1QBP and β-actin expression in 786-0, 786-0-scr and C1QBP shRNA-treated 786-0 cells were analyzed by western blotting. (**C**) After C1QBP knockdown, transcription of genes related to different cellular functions were characterized. (**D**) To confirm the finding of microarray, real-time PCR were performed, two downregulated (CLDN4, MPZL3) and eight upregulated (Rnd-3, Cadherin-11, CTGF, L1CAM, CCDC80, hFat, IL-8 and Del1) genes were consistent with the microarray results. (**Indicate *P* < 0.01).

To investigate the function of C1QBP in RCC cells, we used 786-0 cells with lentivirus-mediated knockdown of C1QBP. Western blotting and real-time PCR were used to determine the expression of C1QBP in 786-0, 786-0-scr and 786-0-shC1QBP cells. Real-time PCR indicated that the expression of C1QBP in 786-0-shC1QBP cells was significantly decreased than 786-0 and 786-0-scr cells (Fig. 1A). Moreover, western blotting confirmed the reduction of C1QBP protein expression (Fig. 1B). Thus we established stable C1QBP knockdown RCC cell line.

We then prepared total RNA which was subjected to microarray analysis. After normalizing the gene expression, we assessed the profile of gene expression in 786-0, 786-0-scr and 786-0-shC1QBP stable cells and set the threshold of differential expression at 2-fold and obtained a list of genes whose expression was related to the C1QBP level. GO analysis of these genes clearly showed that C1QBP knockdown affected expression of many genes, including those for cell adhesion, acute and chronic inflammatory response, cell motion, cell cycle, cell proliferation, as well as post-translational modification (Supplementary Figs 1 and 2 and Fig. 1C).

We chose 8 up-regulated genes (Rnd-3, Cadherin-11, CTGF, L1CAM, CCDC80, hFat, IL-8 and Del1) and 3 down-regulated genes (C1QBP, CLDN4, MPZL3) for further analysis with a different analytical platform (Supplementary Table 1). Real-time PCR analysis with RNA from cells of 786-0-scr and 786-0-shC1QBP indicated that the expression of two downregulated (CLDN4, MPZL3) and eight upregulated (Rnd-3, Cadherin-11, CTGF, L1CAM, CCDC80, hFat, IL-8 and Del1) genes were consistent with the microarray results. Among these genes, we focused on L1CAM whose expression was most significantly up-regulated in the 786-0-shC1QBP cells (Fig. 1D).

### C1QBP regulates adhesion and invasion of RCC cell

**Figure 2:**
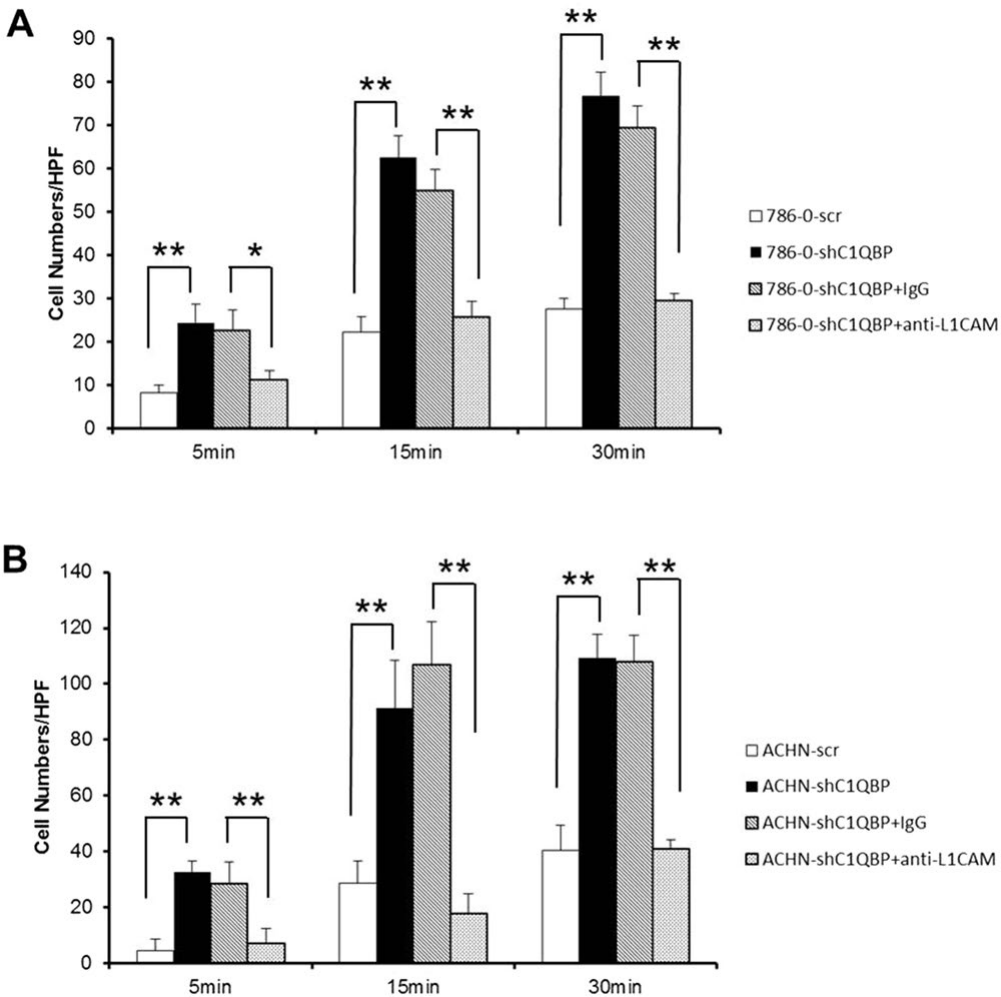
C1QBP regulates the adhesion ability of RCC cells. (**A**) shC1QBP regulated L1CAM to promote the cell adhesion ability of 786-0 cell. Depletion of C1QBP increased 786-0 cell adhesion ability and it was reversed by using L1CAM antibody. (**B**) shC1QBP regulated L1CAM to promote the cell adhesion ability of ACHN cell. Depletion of C1QBP increased ACHN cell adhesion ability and it was reversed by using L1CAM antibody (20 μg/ml) (Sigma Aldrich, St. Louis, MO, USA). Cell adhesion capacity were measured in 5 min, 15 min and 30 min after cell seeding, cell numbers in five fields were counted for each coverslip under the microscope (*Indicate *P* < 0.05, **indicate *P* < 0.01).

Using cell adhesion assay, we examined the impact of knocking down C1QBP on RCC cell adhesion. We found that cell adhesion ability of 786-0-shC1QBP cells was significantly increased compared with that in 786-0-scr especially at 15 min and 30 min after cell seeding. Importantly, addition of L1CAM antibody could reverse the shC1QBP-increased 786-0 cell adhesion (Fig. 2A). Conventional cell adhesion assay was also used to confirm this conclusion (Supplementary Fig. 4A). Consistently, addition of C1QBP inhibited the 786-0 cell adhesion (Supplementary Fig. 4B). This result indicated that C1QBP knockdown significantly increased adhesion ability of 786-0 cell likely through increase of L1CAM expression. Similar effects of C1QBP were further confirmed in ACHN cells (Fig. 2B and Supplementary Fig. 4C).

**Figure 3:**
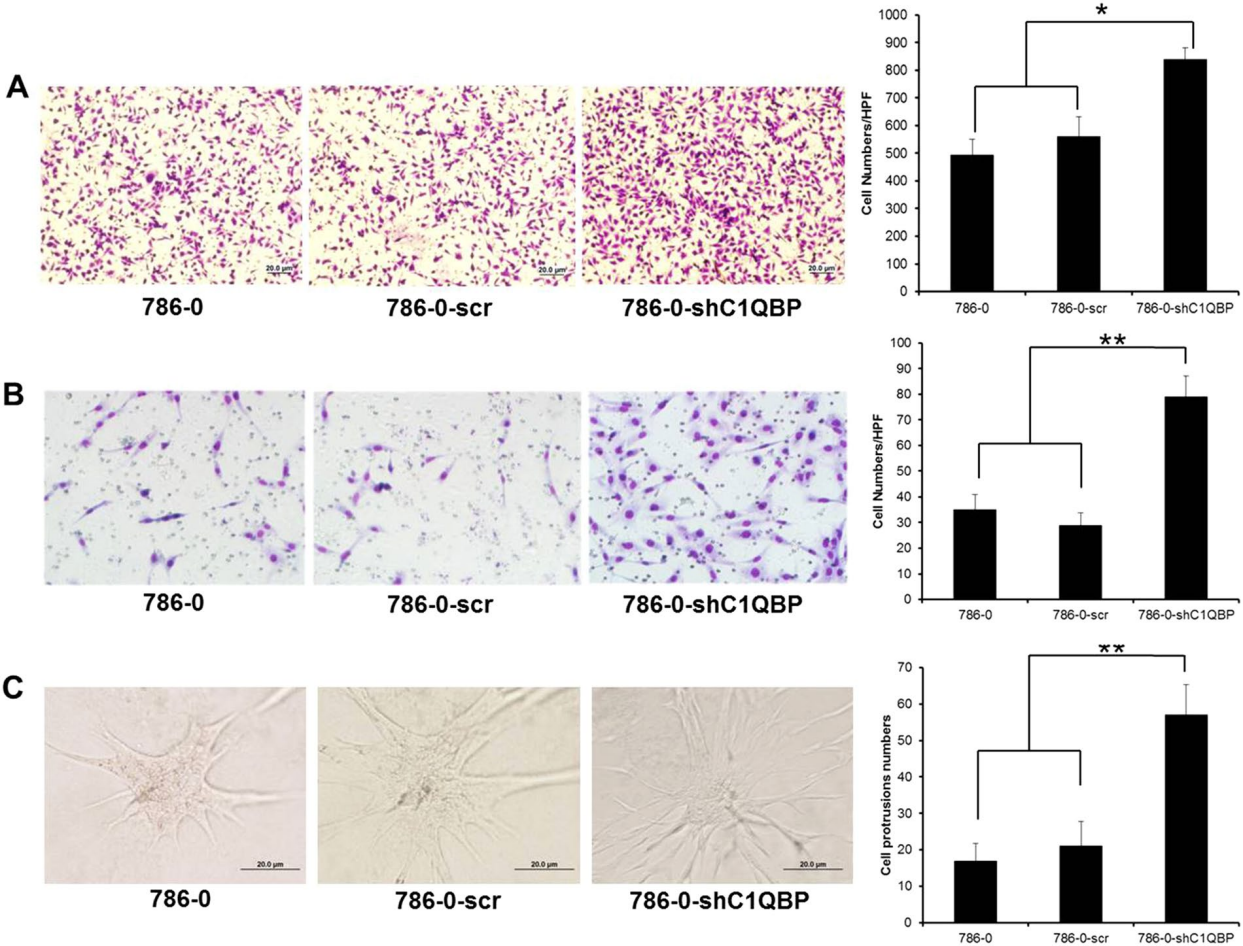
C1QBP regulates invasion of RCC cells. (**A**) Depletion of C1QBP increased cell migration ability of 786-0 cells. Left: Representative microscopic photographs of the cells. Right: Quantitation of cell migration. (**B**) Depletion of C1QBP increased cell invasion ability of 786-0 cell. Left: Representative microscopic photographs of the cells. Right: Quantitation of cell invasion. (**C**) 3D spheroid invasion assay was performed in 786-0 cells transfected with scramble or sh-C1QBP. Left: Representative microscopic photographs of the cells. Right: Quantitative analysis (cell spheroid protrusions numbers were counted in six randomly chosen microscopic fields per membrane). (*Indicate *P* < 0.05, **indicate *P* < 0.01).

The L1CAM is a cell adhesion protein thus likely influencing cell movement. Since cell motility is an essential part of cancer metastasis, we performed a transwell assay in 786-0 and highly metastatic ACHN cells with different expressions of C1QBP (Fig. 1A,B and Supplementary Fig. 3). The results showed that the migratory capacity of 786-0-shC1QBP cells was significantly increased compared to the control group (Fig. 3A). Likewise, enhanced cell migration after C1QBP knockdown was also observed in ACHN cells (Supplementary Fig. 5B). As expected, addition of C1QBP in 786-0 and ACHN cells resulted in decreased cell migration compared with the control cells (Supplementary Fig. 5A and C). Using matrigel-coated transwell invasion assay, we found knockdown of C1QBP in 786-0 cells promoted cell invasion (Fig. 3B). Similar results were obtained when we replaced the matrigel-coated transwell invasion assay with another 3D culture invasion assay showing C1QBP knockdown led to drastically increased cell protrusions in 786-0 cells (Fig. 3C).

Taken together, results from Figures 2 and 3 and supplementary Figures 4 and 5 demonstrated that C1QBP plays a negative role in RCC cell adhesion, migration and invasion.

### C1QBP regulates GSK3/β-Catenin /L1CAM signaling to influence the RCC cell adhesion and invasion

**Figure 4:**
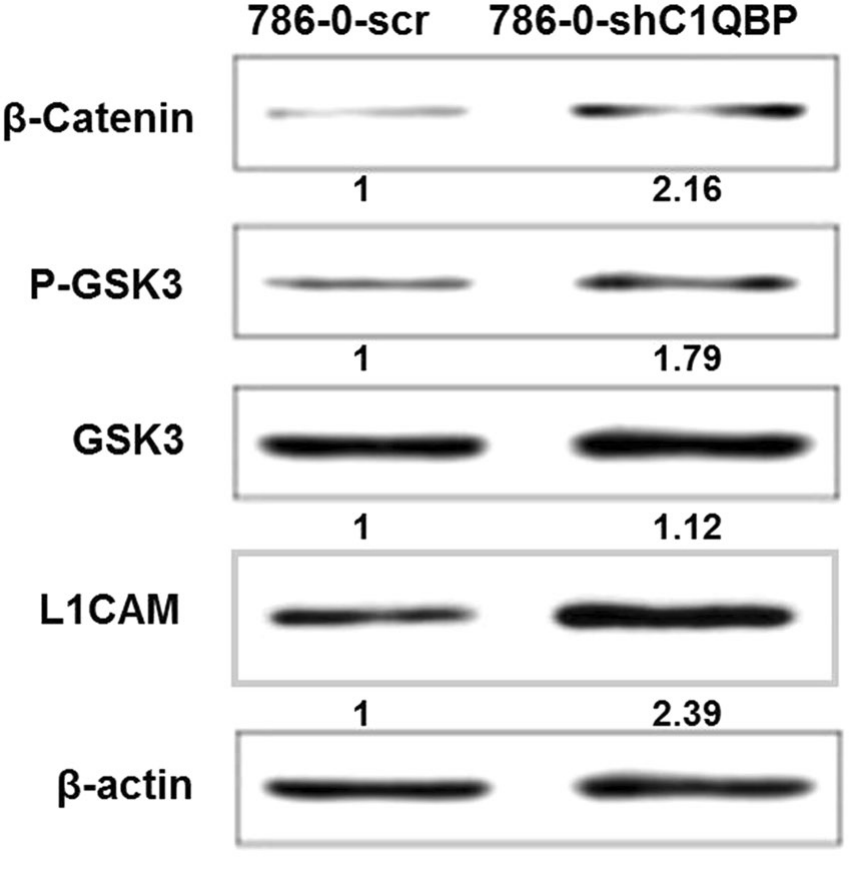
The expression of associated proteins after C1QBP knockdown. Cells lysis were collected and quantified by BCA assay to ensure equal protein loading for western blotting. β-Catenin, p-GSK3, GSK3 and L1CAM were measured. Experiment was repeated 3 times.

β-Catenin is an important intracellular signaling molecule associated with cell anchorage^12^. After C1QBP knockdown in 786-0 cell, western blotting was performed to test the expression of β-Catenin, GSK-3β, p-GSK-3β and L1CAM. The result showed that C1QBP silencing induced GSK-3β phosphorylation and the expression of β-Catenin and L1CAM (Fig. 4 and Supplementary Fig. 6). Our data suggested that C1QBP silencing promoted 786-0 cell adhesion and invasion by increasing GSK-3β phosphorylation and its inactivation with a consequent increase of β-Catenin and L1CAM.

### C1QBP regulates metastasis of RCC cell *in vivo*

**Figure 5:**
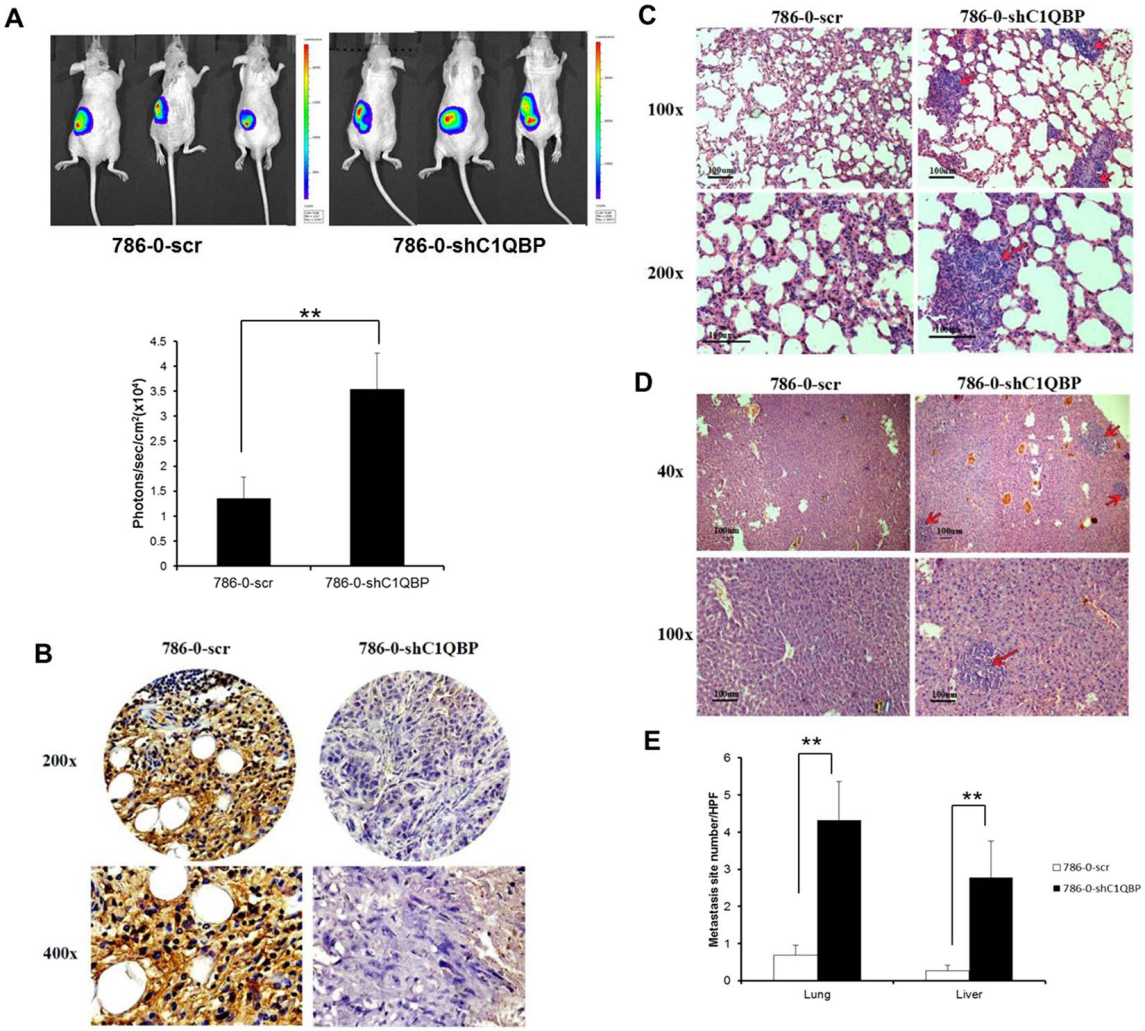
*In vivo* mouse model to test the effects of C1QBP on RCC metastasis. Luc-786-0 (786-0-scr) and luc-786-0-shC1QBP (786-0-shC1QBP) cells were implanted into nude mice left kidney. The reporter gene signals were detected by IVIS imaging system. (**A**) The tumor growth was detected by IVIS system. Below: Quantitation of the tumor sizes by the luciferase bioluminescence intensity. (**B**) Mouse tumor samples were collected for IHC staining for C1QBP. (**C**) HE staining for the lung metastasis. (**D**) HE staining for the liver metastasis. (**E**) Quantification of the tumor metastasis in lung and liver. (**Indicate *P* < 0.01).

To confirm the above *in vitro* results in cell lines in an *in vivo* mouse model, we performed the orthotopic implantation of RCC 786-0 cells stably transfected with firefly luciferase and shC1QBP expression into the subrenal capsule of left kidney of nude mice. The primary tumors (Fig. 5A) and metastatic tumors (Fig. 5C,D) were monitored. The results revealed that mice with a knocked-down C1QBP in 786-0 cells (Fig. 5B) had more metastatic foci detected in lung and liver (Fig. 5C–E), indicating that loss of C1QBP can enhance RCC metastasis thus C1QBP could negatively regulate RCC metastasis *in vivo*.

## Discussion

RCC is one of the most common malignant tumors in the urinary system. The treatment of RCC has changed greatly over the past 15 years. Progress in the surgical management of the primary tumor and increased understanding of the molecular biology and genomics of the disease have led to the development of new therapeutic agents^13^. The most common subtype of RCC is clear cell RCC, which comprises about 75% of RCCs in surgical series^14^. Because of the complicated mechanisms of tumor development, there’s much to explore for development of potential targeted therapies for RCC as well as discovery of new biomarkers for RCC staging and prognosis. In the previous study^10^, immunohistochemistry and western blotting are used to study the expression of C1QBP in renal carcinoma tissue and cells, the expression of C1QBP in renal carcinoma tissue is lower compared with para-carcinoma tissue. We further examined the potential biological consequence of a lowered expression of C1QBP in this study.

Progression from the primary tumor to distant metastasis is associated with changes in different cellular properties which include the misregulation of cell-cell and cell-extracellular matrix (ECM) adhesion^15^. The adhesions are associated with a variety of cell adhesion factors and play an important role in different developmental stages of the tumor. If activation of these factors is inhibited, the cell adhesion and metastatic abilities will also be inhibited. In this study gene microarray was used to compare the gene expression profiling of 786-0 cells with that of C1QBP knockdown, a set of differentially expressed genes associated with cell adhesion were identified. Further functional assays indicated that C1QBP knockdown could increase cell adhesion and migration ability of RCC cell likely through upregulation of L1CAM protein.

**Figure 6:**
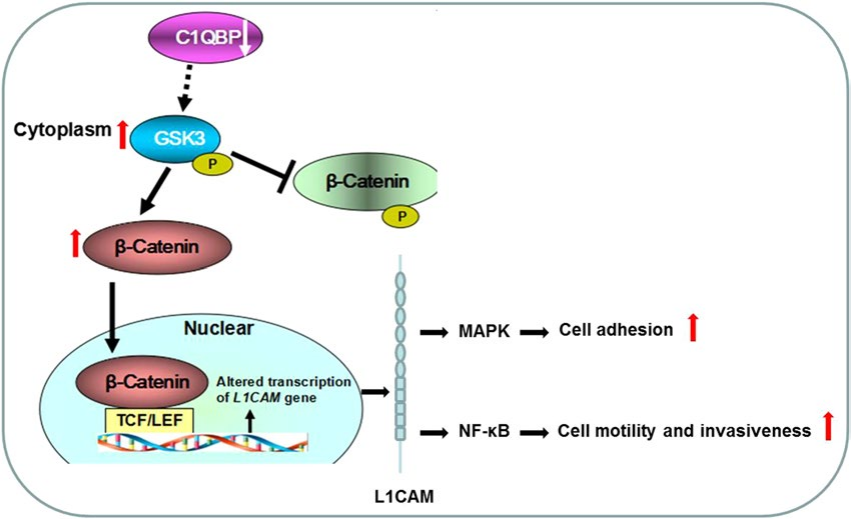
A schema illustrating the mechanism of C1QBP regulates GSK3/β-Catenin/L1CAM signaling in RCC.

L1CAM, a 200–220 kDa transmembrane glycoprotein of the immunoglobulin (Ig) superfamily^16, 17^, is discovered as a cell adhesion molecule and plays an essential role in the development of the nervous system^18^. Moreover, L1CAM is also highly relevant for the progression of human tumors^17^. L1CAM was recently identified as a target gene of the β-Catenin-TCF signaling in colorectal cancer cells^19^ and β-Catenin can activate L1CAM transcription^20^. β-Catenin phosphorylation by GSK3 can result in its degradation through ubiquitin-mediated mechanism and GSK3 auto-phosphorylation inactivates its activity. Meanwhile, β-Catenin could translocate to the nucleus to regulate downstream genes such as L1CAM. Indeed, we found that C1QBP knockdown resulted in GSK3 phosphorylation and an increasing of  β-Catenin in RCC cells, therefore stimulated RCC cell adhesion, migration as well as metastasis (Fig. 6).

L1CAM is a static cell adhesion molecule in the nervous system. Its ability to promote motility of neuronal cells is essential for the development or remodeling of the nervous system^21^. These observations point to a switch from a static to a dynamic L1CAM function. Here we showed that L1CAM not only promoted static cell-cell adhesion that keeps tumor cells together, but also RCC cell migration and invasion. Recent data indeed suggested a number of possibilities such as (1) L1CAM homophilic interactions promote static cell-cell binding and trigger predominantly the MAPK pathway^22, 23^. (2) The cleavage of extracellular L1CAM domain and binding of integrins to RGD site triggers NF-κB activation and renders cells more motile and invasive^24, 25^. These different modes of signaling could be linked to the different functions of L1CAM either as a static adhesion or a motility-promoting molecule. The different signaling mechanisms could translate into the activation of different kinases that phosphorylate L1CAM and thereby play different role in different tumor stage.

In conclusion, C1QBP may regulate L1CAM expression in RCC cell through the Wnt/β-Catenin pathway, thus affecting RCC cell adhesion, migration and metastasis.

## Electronic supplementary material

Supplementary Information file
