# Supplementary material for: C1QBP suppresses cell adhesion and metastasis of renal carcinoma cells
Source: Sci Rep. 2017 Apr 20;7:999. doi: 10.1038/s41598-017-01084-w (PMC5430506; doi:10.1038/s41598-017-01084-w)
Supplement: Supplementary file 1 — Supplementary Information file [file 41598_2017_1084_MOESM1_ESM.pdf]

C1QBP suppresses cell adhesion and metastasis of renal carcinoma cells

Yong Wang [a], Donghe Fu [b], Jing Su [b], Yajing Chen [b], Can Qi [c], Yin Sun [d], Yuanjie Niu [a], Ning Zhang [e*], Dan Yue [b*]

[a] *Department of Urology, Tianjin Institute of Urology, Tianjin Medical University Second Hospital, Tianjin 300211, China*

[b] *School of Laboratory Medicine, Tianjin Medical University, Tianjin 300203, China*

[c] *Department of Urology, Children's Hospital of Hebei Province, Shijiazhuang 050031, China*

[d] *Department of Radiation Oncology, University of Rochester Medical Center, Rochester, NY, 14642, USA*

[e] *Tianjin Medical University Cancer Institute and Hospital, National Clinical Research Center for Cancer, Key Laboratory of Cancer Prevention and Therapy; Research Center of Basic Medical Sciences; Tianjin 300070, China*

* Corresponding authors.

Dan Yue

School of Laboratory Medicine, Tianjin Medical University, Tianjin 300203, China

E-mail: danyue0705@sina.cn

Ning Zhang

Tianjin Medical University Cancer Institute and Hospital, National Clinical Research Center for Cancer, Key Laboratory of Cancer Prevention and Therapy; Research Center of Basic Medical Sciences; Tianjin 300070, China

E-mail: zhangning@tijmu.edu.cn

Supplementary Method

Conventional cell adhesion assay

For cell adhesion assays, 96-well tissue culture plates were coated with 5 μg/ml fibronectin in PBS at 37 ℃ for 1 h, and then covered with serum free DMEM containing 2% BSA for another 1 h. Cells were detached and plated in triplicate onto the fibronectin-coated wells at 2.5×10^5 cells per well. Cells were allowed to adhere to the fibronectin-coated surface for 20 and 40 min, followed by four intensive washes with DMEM to remove non-adherent cells, and then incubated in 5 μg/ml MTS in complete medium at 37 ℃ for 1 h. The absorbance was measured on a Biotek ELx800 spectrophotometer at 490 nm. Values for the triplicate wells were divided by the corresponding cell number standard value to yield relative A490, which were subsequently normalized to the average of the control for comparison purposes.

Supplementary Figure legend

Supplementary Figure 1

GO analysis of genes expression change in 786-0-shC1QBP cells.

Supplementary Figure 2

The top-ranked canonical pathways associated with C1QBP knockdown in 786-0 cells

Supplementary Figure 3

Screening the expression of C1QBP.

(A) C1QBP and β-actin expression in control group and C1QBP overexpression 786-0 cells were analyzed by western blotting. (B) C1QBP and β-actin expression in control group and C1QBP overexpression ACHN cells were analyzed by western blotting. (C) C1QBP and β-actin expression in ACHN, ACHN-scr and C1QBP shRNA-treated ACHN cells were analyzed by western blotting.

Supplementary Figure 4

C1QBP regulates adhesion of renal cell carcinoma cells

(A) shC1QBP regulated L1CAM to promote the cell adhesion ability of 786-0 cell. Depletion of C1QBP increased 786-0 cell adhesion ability and it was reversed by using L1CAM antibody. Cell adhesion capacity were measured in 20min and 40min. (B) Overexpression of C1QBP decreased 786-0 cell adhesion ability. (C) Overexpression of C1QBP decreased ACHN cell adhesion ability. Cell adhesion capacity were measured in 5min, 15min and 30min, cell numbers in five fields were counted for each coverslip under microscopy (*indicate $P<0.05$, **indicate $P<0.01$).

Supplementary Figure 5

C1QBP regulates migration of renal cell carcinoma cells

(A) Overexpression of C1QBP increased 786-0 cell migration ability. (B) Depletion of C1QBP increased ACHN cell migration ability. (C) Overexpression of C1QBP decreased ACHN cell migration ability. (* indicate $P<0.05$).

Supplementary Figure 6

The expression of associated proteins after C1QBP knock down in ACHN cell.

Cells lysis were collected and quantified by BCA assay to ensure equal protein loading for Western blotting. β-Catenin, p-GSK3, GSK3 and L1CAM were measured.

Supplementary Figure

Supplementary Figure 1

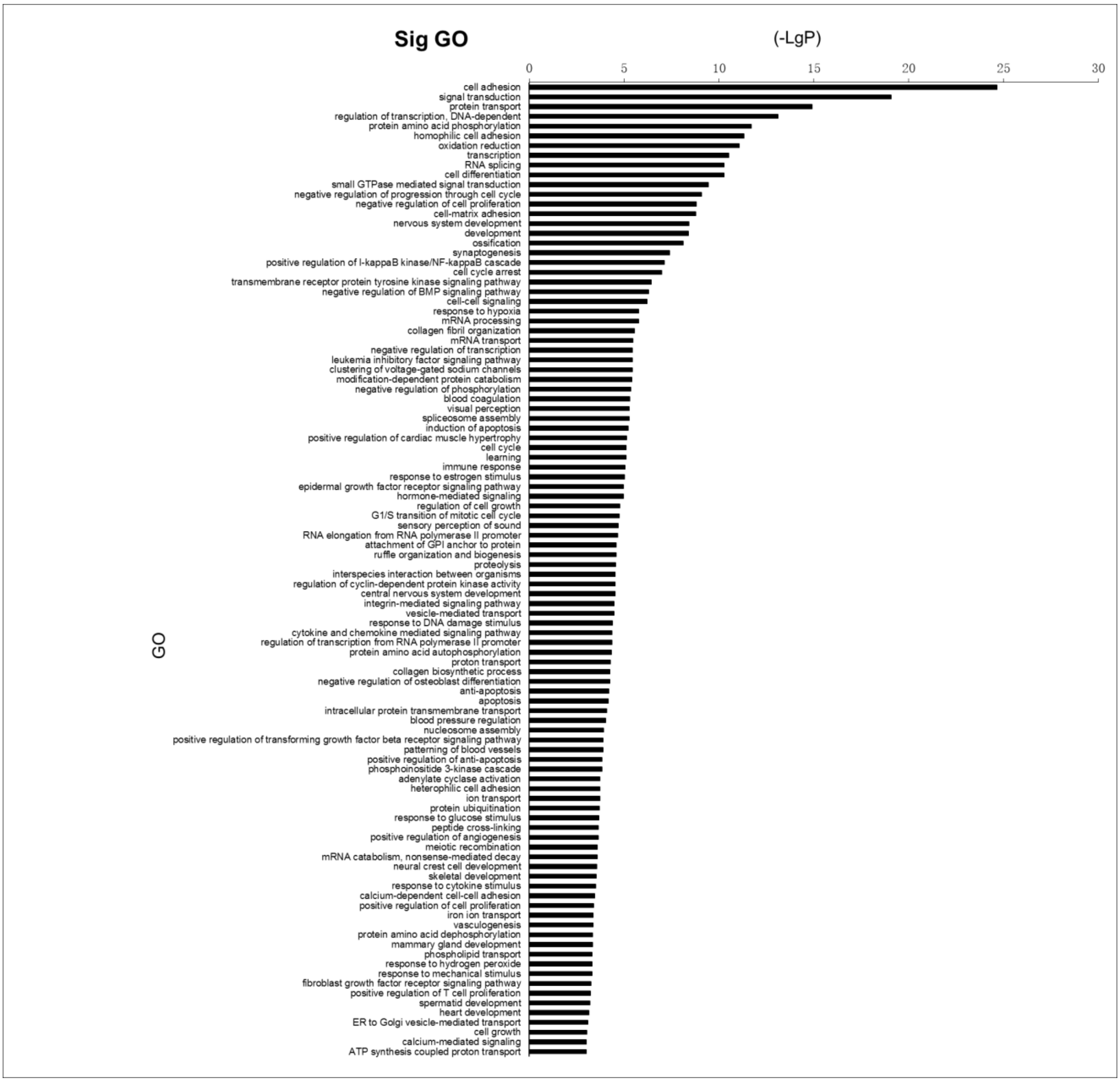

Supplementary Figure2

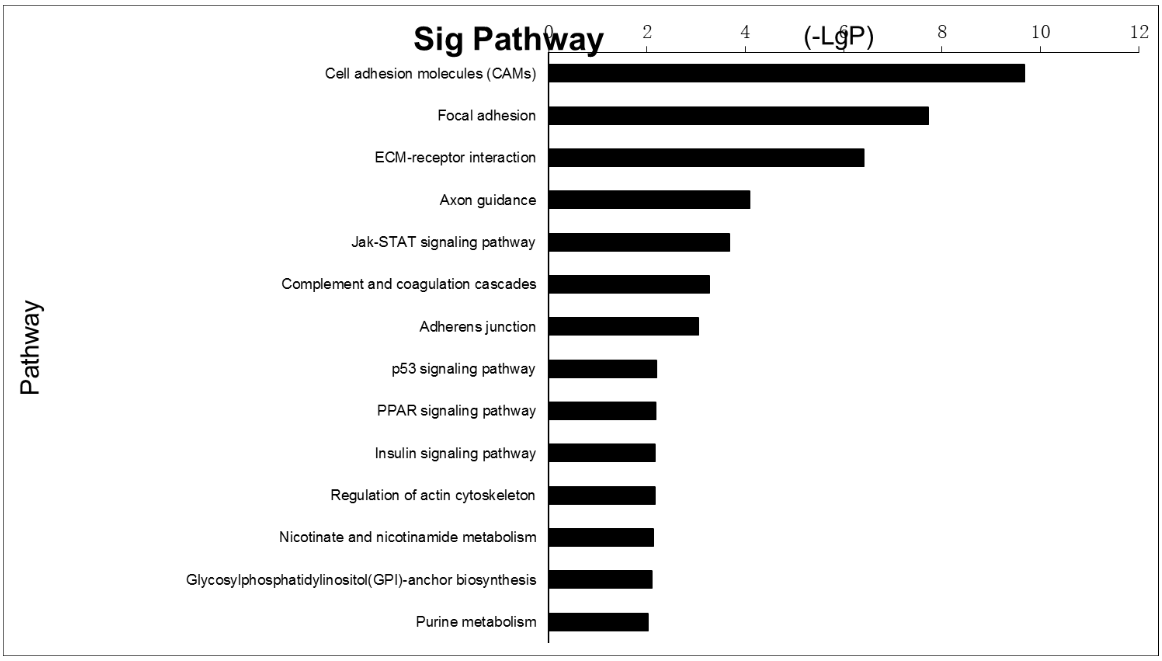

Supplementary Figure3

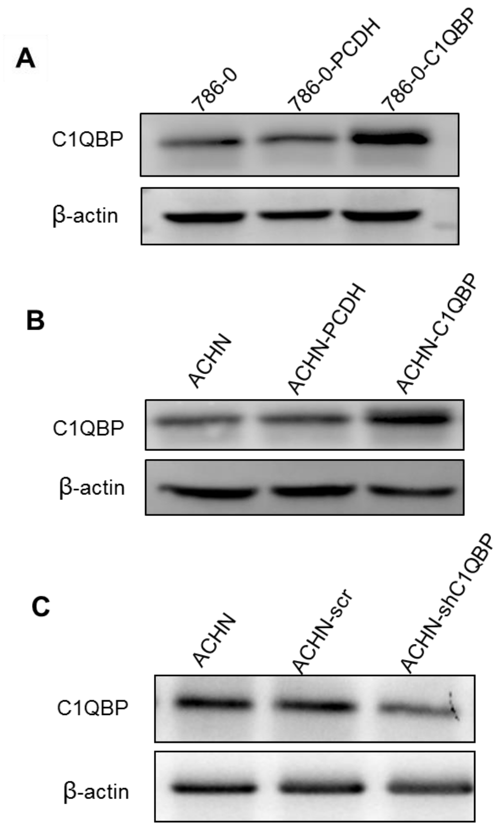

Supplementary Figure 4

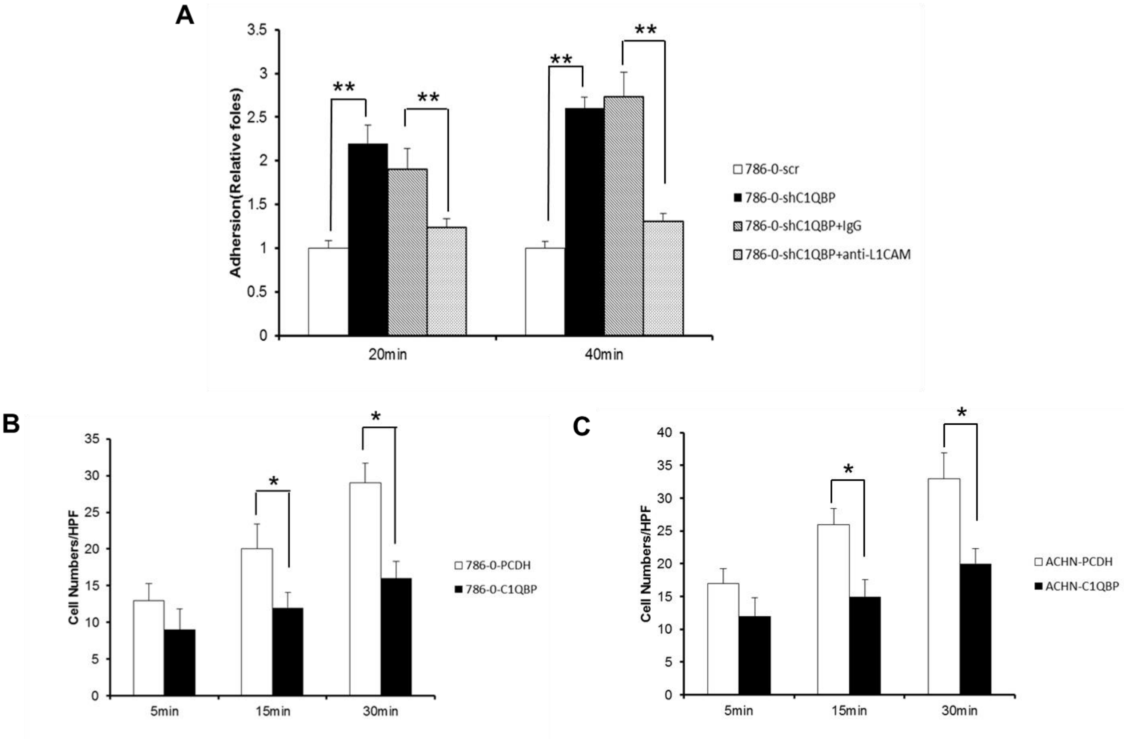

Supplementary Figure 5

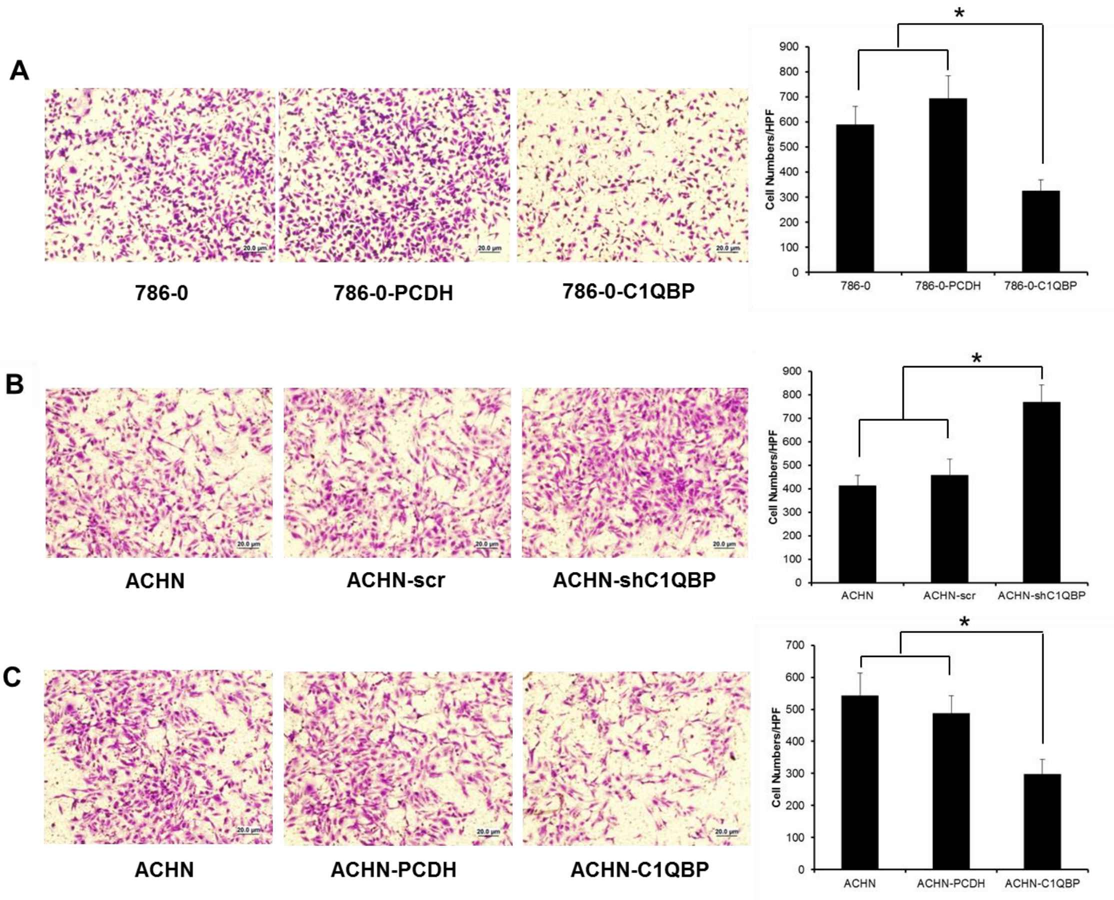

Supplementary Figure 6

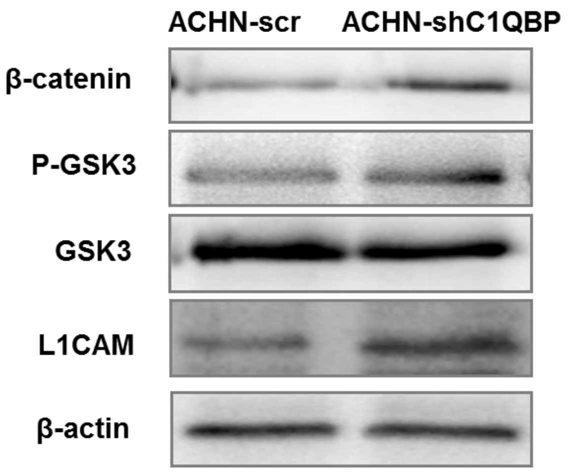

Supplementary Table

Supplementary Table 1: Up-regulated and down-regulated genes in the C1QBP knockdown 786-0 cell

Entrez Gene	Gene Symbol	Gene Title	RefSeq Transcript ID	Fold Change
151887	CCDC80	coiled-coil domain containing 80	NM_199511 /// NM_199512	2.19446
1009	CDH11	cadherin 11, type 2, OB-cadherin (osteoblast)	NM_001797	2.48538
1364	CLDN4	claudin 4	NM_001305	-3.03641
1490	CTGF	connective tissue growth factor	NM_001901	2.22361
10085	EDIL3	EGF-like repeats and discoidin I-like domains 3	NM_005711	2.09133
79633	FAT4	FAT tumor suppressor homolog 4 (Drosophila)	NM_024582	2.10762
3576	IL8	interleukin 8	NM_000584	-2.11904
3897	L1CAM	L1 cell adhesion molecule	NM_000425 /// NM_001143963 /// NM_024003	2.71435
196264	MPZL3	myelin protein zero-like 3	NM_198275	-2.00527
390	RND3	Rho family GTPase 3	NM_005168	2.03141
